# Reply to: A comparative study of diagnostic accuracy in 3026 pleural biopsies and matched pleural effusion cytology with clinical correlation: A methodological issue

**DOI:** 10.1002/cam4.5524

**Published:** 2022-12-07

**Authors:** Ivan K. Poon, Ronald C. K. Chan, Joseph S. H. Choi, Joanna K. M. Ng, Katsie T. Tang, Yolanda Y. H. Wong, Ka Pang Chan, Wing Ho Yip, Gary M. Tse, Joshua J. X. Li

**Affiliations:** ^1^ Department of Anatomical and Cellular Pathology, Prince of Wales Hospital The Chinese University of Hong Kong Hong Kong; ^2^ Department of Medicine and Therapeutics, Prince of Wales Hospital The Chinese University of Hong Kong Hong Kong

We thank Zhang and colleagues for their readership and letter.^1^, ^2^ Zhang et al. have commented that diagnostic odds ratio (DOR), negative‐to‐positive likelihood ratio (LR), and area under receiver operating characteristic curves (AUC) could serve additional metrics for exploring the diagnostic performance of pleural biopsy and pleural effusion, and that the lack of AUC constitutes methodological insufficiency. We are grateful for their interesting suggestions that merit discussion.

The statistical analyses of our manuscript were based on a five‐tiered biopsy and cytology diagnostic categorization. Two cut‐offs for positive/negative test result were explored, considering malignant diagnoses (B5/C5) only as positive or including the category of suspicious for malignancy (B4/C4) as positive. All subsequent analyses were based on two‐by‐two tables of pleural biopsies and pleural effusion cytology specimens. Sensitivity (Sn), specificity (Sp), positive predictive value (PPV), negative predictive value (NPV), accuracy, and risk of malignancy (ROM) between biopsy and cytology, and subgroup stratified by disease types were compared.

Our study addressed the difference in diagnostic performance of biopsy versus cytology, as such, only paired specimens were included. Statistics were selected for comparing two‐by‐two tables, we regarded the most direct metrics, namely Sn, Sp, PPV, NPV, accuracy and ROM sufficient for the purpose. It is possible to extrapolate the DOR and LR from the raw data. However, as the majority of biopsies and cytology specimens received in a hospital laboratory are unpaired, the samples included in our study were discontinuous, thus not representative for assessment of overall diagnostic performance. Guidelines and cohorts have established references for these metrics.^3^, ^4^

On the same note, AUC may be more suited for determining thresholds for continuous variables.^5^ The diagnostic categories of B1/C1 to B5/C5 corresponds to 1: insufficient/inadequate, 2: benign, 3: atypia, 4: suspicious, and 5: malignant. These are discrete categories and do not represent a continuous scale for the ROM, in particular for the B1/C1 (insufficient/inadequate) category. As opposed to serological or biological tests, the nature of synthesized pathologic reporting is not compatible with a continuous or numeric scale. A possibility would be to utilize clinical parameters and qualitative cytologic assessment for a predictive multi‐parameter scoring system.^6^, ^7^ Such a scoring system, when validated with a robust AUC, has the potential to improve upon the current five‐tiered diagnostic categories.

The role of effusion cytology is irreplaceable in the investigation of pleural diseases, but not without certain limitations. We highly appreciate the opportunity Zhang and colleagues offered in further deliberation on the issue.

## AUTHOR CONTRIBUTIONS

**Ivan K. Poon:** Writing – original draft (equal). **Ronald C. K. Chan:** Validation (equal). **Joseph S. H. Choi:** Writing – review and editing (equal). **Joanna K. M. Ng:** Validation (equal). **Katsie T. Tang:** Validation (equal). **Yolanda Y. H. Wong:** Validation (equal). **Ka Pang Chan:** Validation (equal). **Wing Ho Yip:** Validation (equal). **Gary M. Tse:** Writing – review and editing (equal). **Joshua J. X. Li:** Writing – original draft (equal).

## CONFLICT OF INTEREST

The authors declare that there is no conflict of interest regarding the publication of this paper.
